# Loss of Ripk3 attenuated neutrophil accumulation in a lipopolysaccharide-induced zebrafish inflammatory model

**DOI:** 10.1038/s41420-022-00891-z

**Published:** 2022-02-26

**Authors:** Wanying Wen, Jiakui Chen, Yuxin Zhou, Gaofei Li, Yiyue Zhang

**Affiliations:** Division of Cell, Developmental and Integrative Biology, School of Medicine, South China University of Technology, Guangzhou, 510006 People’s Republic of China

**Keywords:** Inflammation, Innate immune cells

## Abstract

Neutrophils are important effector cells during inflammation, which play complex roles. Therefore, investigating the regulation of neutrophil accumulation during inflammation might provide targets for treating related diseases. In the present study, we generated a *ripk3*-deficient zebrafish line to study the roles of Ripk3 in neutrophil-related inflammation. The homeostatic hematopoiesis and cytokine expression of the *ripk3*-deficient larvae were unaltered. The *ripk3*-deficient larvae with caudal fin fold injury exhibited similar neutrophil enrichment with wild-type larvae, suggesting that Ripk3 is not essential for non-infectious inflammatory responses. When challenged with lipopolysaccharide (LPS), the *ripk3*-deficient larvae showed significantly less neutrophil accumulation in the injection site and differential expression of several key cytokines. Ripk3 inhibitors could also attenuate neutrophil accumulation in wild-type larvae, indicating that Ripk3 could serve as a candidate target for inflammation treatment. In summary, our study indicated that Ripk3 has an essential role in LPS-induced inflammatory responses. It was suggested that the *ripk3*-deficient zebrafish might be applied in developing infectious disease models, while Ripk3 also has potential as an inflammation-treatment target.

## Introduction

Inflammation is an immune response triggered by biological, physical, and chemical inflammatory factors in various tissues and organs of the body. Inflammation initiates as a short-term acute response that mainly involves leukocyte infiltration into the inflammation area, phagocytosis, and tissue repairment [1]. At the onset of inflammation, sentinel innate immune cells such as macrophages and dendritic cells are activated and start to produce a series of mediators, including cytokines and chemokines [2]. Subsequently, neutrophils, natural killer cells, and monocytes are attracted to the inflammation site by these mediators [3]. During inflammation, an important effector cell type is the neutrophil [4], which plays double-sided roles. Generally, neutrophils play essential anti-pathogen roles during infections, as the impairment of neutrophils recruitment would result in pathogen dissemination and even death [5]. On the other hand, neutrophils also induce local tissue damage at the inflammation loci and even result in organ dysfunction [6, 7]. The activation of neutrophils could even trigger some chronic inflammatory diseases, such as atherosclerosis and type II diabetes [8]. Thus, appropriate activation and suppression of neutrophils are critical for inflammatory progress and homeostasis.

Receptor-interacting serine/threonine protein kinase 3 (RIPK3) is a serine/threonine kinase, considered to be a critical element of necroptosis [9, 10]. Inhibitors targeting RIPK3-dependent necroptosis have shown therapeutic potential for multiple diseases such as cancer and metabolic disorders [11]. Accumulating evidence has established that RIPK3 is also an inflammation regulator, which probably has a non-necrotic function [12]. In bone marrow-derived dendritic cells and aortic smooth muscle cells, RIPK3 plays role in inflammation responses by controlling the activation of NF-κB [13, 14]. Inappropriate RIPK3 activity can lead to primary immunodeficiency and autoinflammatory syndrome due to the activation of the NLRP3 inflammasome [15]. RIPK3 plays an essential role in lipopolysaccharide (LPS)-induced acute inflammatory responses, which are not dependent on caspases [16]. Thus, whether RIPK3 could serve as a potential target to release inflammation is worth further exploration.

Zebrafish is an ideal model for studying inflammatory responses and drug evaluation. In addition to in vitro fertilization, fertility, and easy access to microinjection, the hematopoietic systems between mammals and zebrafish share high conserveness [17], and the inflammation responses of zebrafish are also similar to those of mammals [18]. In zebrafish, Ripk3 was found to be essential in regulating necroptosis [19]. In the type 2 diabetes zebrafish model, Ripk3 contributes to islet inflammation by inducing local IL-1β production and recruiting macrophages [20]. However, the potential role of Ripk3 in regulating neutrophil-associated inflammation has never been reported in zebrafish.

In the present study, we utilize the zebrafish model to study the role of Ripk3 in inflammation and evaluate the potential of targeting Ripk3 in treating inflammation. By generating and further characterizing a zebrafish *ripk3*-deficient line, we found that the *ripk3* deficiency perturbed neutrophil enrichment and affected cytokine expressions upon LPS challenge. We did not find obvious changes in homeostatic hematopoiesis, inflammatory cytokines, and neutrophil accumulation during non-infectious inflammation. Importantly, Ripk3 inhibitors had similar effects to *ripk3*-deficient larvae, indicating that targeting Ripk3 could relieve inflammatory responses. Thus, by using zebrafish as a model, we demonstrate that Ripk3 plays key roles in infectious inflammation and could serve as a therapeutic target for inflammatory-related disease treatment.

## Results

### Knockout of the zebrafish *ripk3* gene by CRISPR-Cas9

**Fig. 1:**
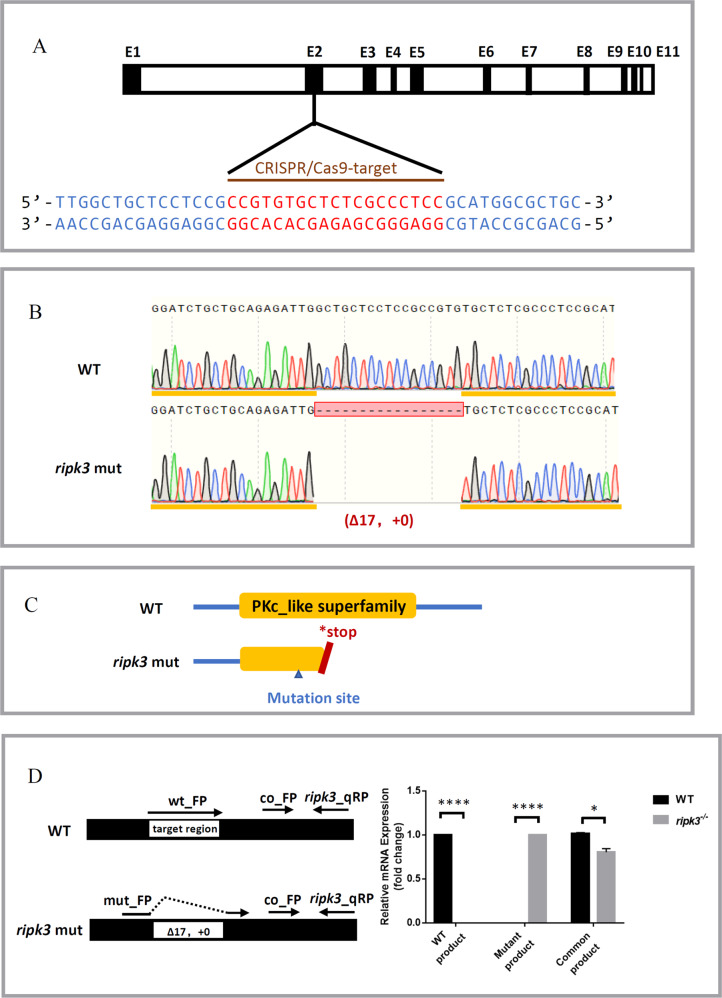
Generation of the *ripk3* knockout zebrafish line. **A** CRISPR target of *ripk3*. The CRISPR/Cas9 target sequence was designed on the second exon of ripk3, and mutations were created using CRISPR-Cas9 technology. **B** Mutation form of *ripk3*-deficient larvae. A mutation strain with 17 bp deletion in the *ripk3* gene (−17 bp, +0) was applied in this study. **C** Protein changes upon *ripk3* mutation. In the *ripk3*-deficient larvae, a premature stop codon was generated and disrupted the PKc_like superfamily protein domain. **D** Validations of the *ripk3* mutation. Q-PCR revealed that the WT or mutant product of *ripk3* mRNA could only be detected in the WT or *ripk3*-deficient larvae, respectively. The expression of the common product showed that *ripk3* was significantly lower expressed in the *ripk3*-deficient larvae (*n* ≥ 10, Student’s *t*-test).

To determine the role of *ripk3* in inflammation, we generated a *ripk3*-deficient zebrafish line by targeting the second exon of *ripk3* with CRISPR-Cas9 technology (Fig. 1A). The F0 individuals were crossed with wild-type (WT) zebrafish to generate F1 heterozygotes, in which a 17 base pair (bp) deletion was found in the *ripk3* gene (−17 bp, +0) (Fig. 1B). The coding of the PKc_like superfamily domain in the Ripk3 protein was predicted to be abolished by a pre-mature stop codon in this mutation (Fig. 1C). To confirm the mutation, the expression levels of *ripk3* mRNA were detected with Q-PCR in WT and *ripk3*-deficient larvae, which showed that the expression of *ripk3* was decreased in *ripk3*-deficient larvae (Fig. 1D, common products). Specific Q-PCR forward primers for WT and mutant *ripk3* genes were designed (Mut_FP and WT_FP) (Fig. 1D and Table S1). The expression levels of mutant Q-PCR products were almost undetectable in the WT larvae, while those of WT products were also undetectable in the *ripk3*-deficient larvae (Fig. 1D), indicating that the WT form was substituted with the mutant form of mRNA in the *ripk3*-deficient larvae. Therefore, the generation of the *ripk3*-deficient line (−17 bp, +0) was successful and confirmed to be a loss-of-function mutation.

### Deficiency of *ripk3* does not affect hematopoiesis

**Fig. 2:**
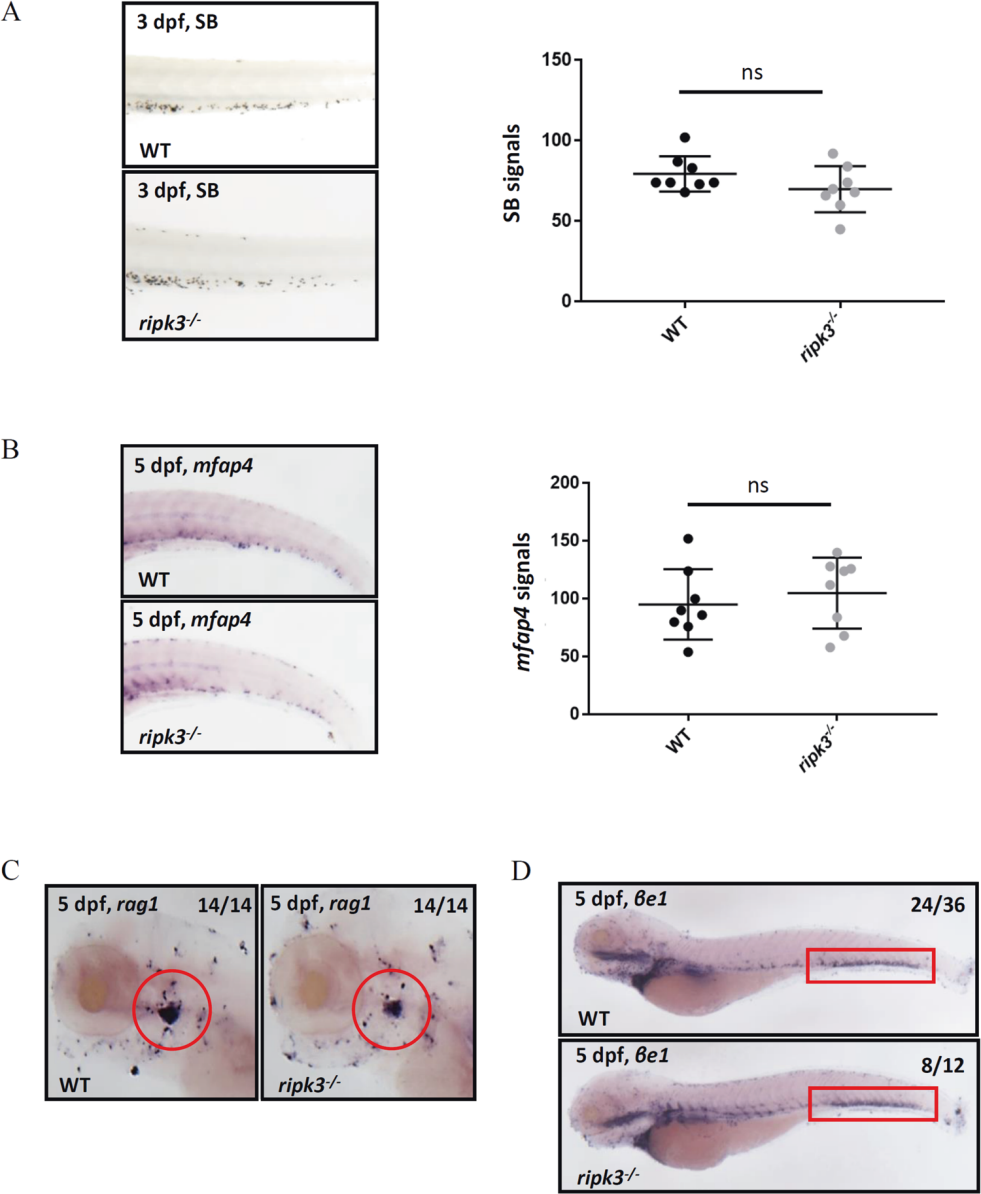
Hematopoiesis was unaltered in *ripk3*-deficient larvae. **A** SB staining showing unchanged neutrophils (*n* = 8, Student’s *t*-test). **B** WISH of *mfap4* showing unchanged macrophages (*n* = 8, Student’s *t*-test). **C** WISH of *rag1* showing normal T lymphocytes (red circle showing the thymus) (Fisher’s exact tests, *n* = 14). **D** WISH of *βe1* showing normal erythrocytes (red box showing the caudal hematopoietic tissue) (Fisher’s exact tests, *n* > 12).

To characterize the effects of *ripk3* disruption on homeostatic hematopoiesis, the numbers of myeloid, lymphoid, and erythroid cells in *ripk3*-deficient larvae were detected by Sudan black (SB) staining and whole-mount in situ hybridization (WISH). The result of SB staining showed no significant difference in the population of neutrophils between *ripk3*-deficient larvae and WT larvae at 3 days post fertilization (dpf) (Fig. 2A). Based on previous studies, *mfap4*, *rag1*, and *βe1* were applied as markers of macrophages, lymphocytes, and erythrocytes, respectively [21–23]. According to the WISH results, there was no significant difference in the population of macrophages, lymphocytes, and erythrocytes between the *ripk3*-deficient larvae and WT larvae at 5 dpf (Fig. 2B–D). Thus, the *ripk3* deficiency had no significant effect on the homeostatic hematopoiesis in zebrafish.

### Deficiency of *ripk3* does not affect cytokines expression

**Fig. 3:**
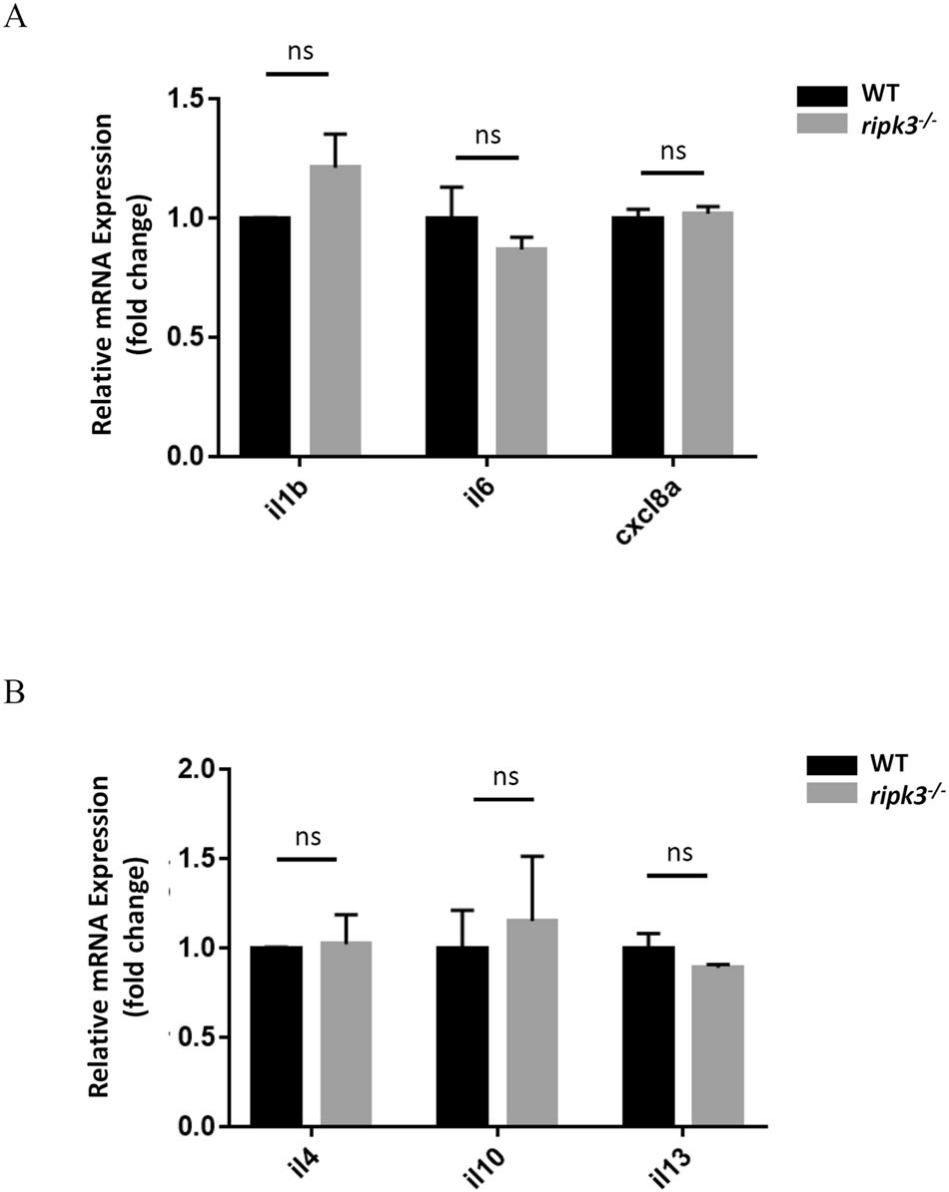
The cytokine expressions kept unchanged in *ripk3*-deficient larvae. **A** Normal pro-inflammatory cytokines expression upon *ripk3* deficiency. The expression levels of *il1b*, *il6*, and *cxcl8a* were unchanged in 3 dpf *ripk3*-deficient larvae (*n* ≥ 10, Student’s *t*-test). **B** Normal anti-inflammatory cytokines expression upon *ripk3* deficiency. The expression levels of *il4*, *il10*, and *il13* were unchanged in 3 dpf *ripk3*-deficient larvae (*n* ≥ 10, Student’s *t*-test).

To examine whether the *ripk3* deficiency would affect inflammation status, we detected the expression levels of several pro-inflammatory (*il1b*, *il6*, and *cxcl8a*) [24] and anti-inflammatory (*il4*, *il10*, and *il13*) [25] cytokines by Q-PCR. The expression of all these cytokines was unaltered upon *ripk3* deficiency compared with their WT controls (Fig. 3A, B). The results demonstrated that the *ripk3* deficiency does not affect the basal expression of cytokines during normal physiological conditions.

### Deficiency of *ripk3* had limited effects on non-infectious inflammatory responses

**Fig. 4:**
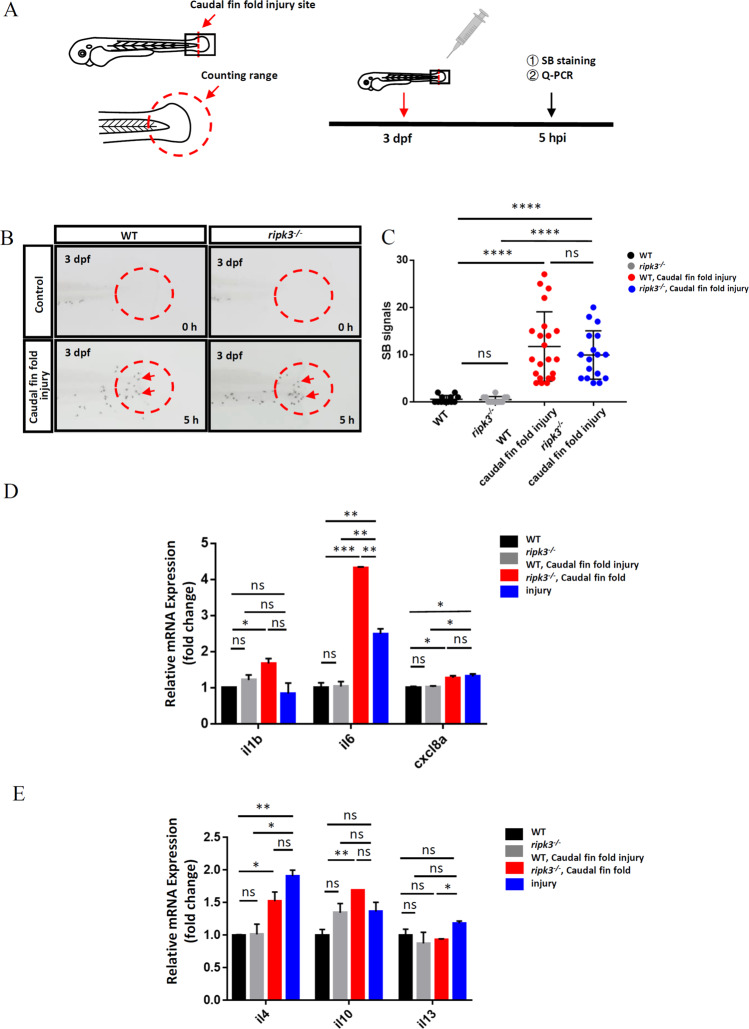
The *ripk3* deficiency induced limited changes in non-infectious inflammation. **A** Experimental design. The caudal fin folds of 3 dpf larvae were injured by sterile syringe needles, and the local accumulation of neutrophils and cytokines expression were detected at 5 hpi. **B**, **C** Normal neutrophils attraction towards the injury site. At 0 hpi, few neutrophils could be found in the injury area (red circle). At 5 hpi, similar numbers of neutrophils (red arrows pointed) were attracted to the caudal fin folds (*n* ≥ 12, Student’s *t*-test). **D** The expression levels of pro-inflammatory cytokines (*il1b*, *il6*, and *cxcl8a*) (*n* ≥ 10, One-way ANOVA). **E** The expression levels of anti-inflammatory cytokines (*il4*, *il10*, and *il13*) (*n* ≥ 10, one-way ANOVA).

Caudal fin fold injury is a well-established methodology to induce non-infectious inflammatory responses in zebrafish [26]. WT larvae first received an injury (Fig. 4A) and were then collected at different time points to optimize the most robust inflammation response. SB staining revealed that increasing neutrophils migrated to the injury transection site until 5 h post-injury (hpi), and neutrophils exited the caudal fin fold after 5 hpi (Fig. S1). Therefore, we applied the caudal fin fold injury to *ripk3*-deficient larvae and collected the samples at 5 hpi to detect their inflammatory responses (Fig. 4A). It was found that both *ripk3*-deficient and WT larvae exhibited almost no neutrophil at the caudal fin fold region before treatment, while similar neutrophil accumulation was found at 5 hpi with no significant difference (Fig. 4B, C). Therefore, in non-infectious inflammatory responses, the *ripk3* deficiency does not affect the recruitment of neutrophils.

We also detected the expression of cytokines through Q-PCR. It was found that the untreated *ripk3*-deficient and WT larvae shared similar cytokine expression levels (Fig. 4D, E), which was identical with the basal expression data (Fig. 3). Upon caudal fin fold injury, the expression levels of all detected pro-inflammatory cytokines (*il1b*, *il6*, and *cxcl8a*) were upregulated in WT (Fig. 4D). However, the injury-induced upregulation of *il1b* and *il6* expression is attenuated in the *ripk3*-deficient larvae, whereas the induction of *il-8* shows no differences between WT and *ripk3*-deficient larvae (Fig. 4D), suggesting that the *ripk3* deficiency attenuated some of the pro-inflammatory responses during injury. For the expression of anti-inflammatory cytokines, the injury could induce *il4* upregulation in both WT and *ripk3*-deficient larvae (Fig. 4E). Interestingly, the injury-induced expression of *il10* was significantly blocked in *ripk3*-deficient larvae, while the injury-unaffected expression of *il13* was slightly induced in the *ripk3*-deficient larvae (Fig. 4E), suggesting that injury stimulated anti-inflammatory cytokines might be affected diversely by *ripk3* deficiency.

Therefore, the above results showed that the deficiency of *ripk3* affected the expression of several cytokines upon non-infectious inflammation, with little effect on the recruitment of neutrophils.

### Deficiency of *ripk3* attenuates LPS-induced inflammatory responses

**Fig. 5:**
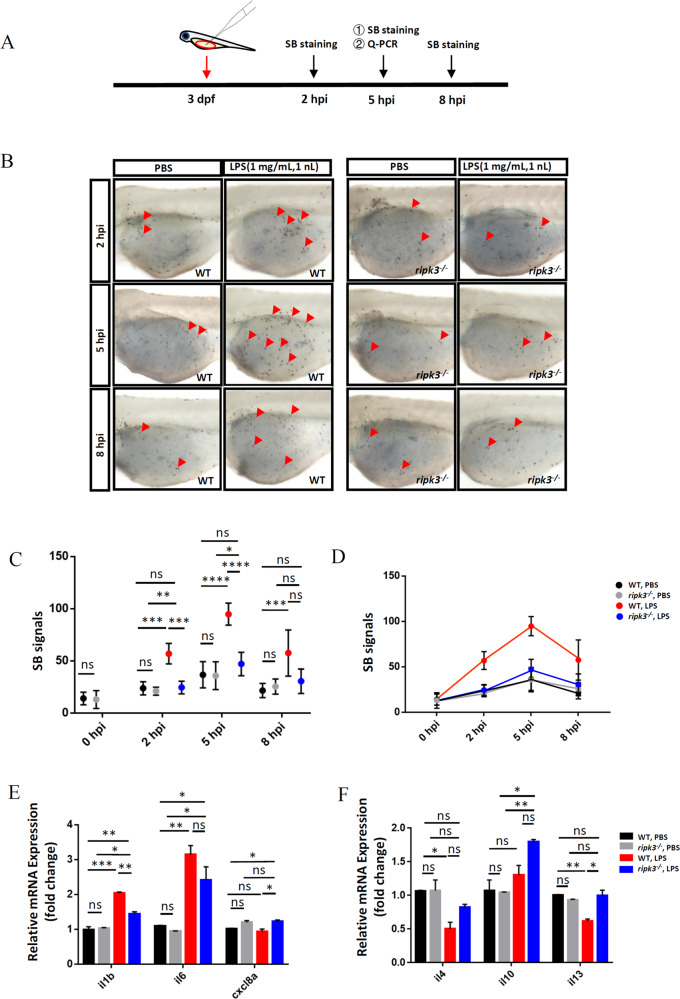
Deficiency of *ripk3* attenuated the LPS-induced inflammatory responses. **A** Experimental design. The 3 dpf WT and *ripk3*-deficient larvae were injected with LPS, and collected at 5 hpi for the detection of cytokines. The neutrophil accumulation was evaluated by SB staining at 2, 5, and 8 hpi. **B** Fewer neutrophils migrated to the injection sites. At 2, 5, and 8 hpi, fewer neutrophils (red arrows pointed) could be found in the yolk sac of the *ripk3*-deficient larvae. **C** Quantification of (**B**) (*n* ≥ 6, one-way ANOVA). **D** Quantification of (**B**), showing the trends of the accumulated neutrophils. **E** The expression levels of pro-inflammatory cytokines (*il1b*, *il6*, and *cxcl8a*) at 5 hpi (*n* ≥ 10, one-way ANOVA). **F** The expression levels of anti-inflammatory cytokines (*il4*, *il10*, and *il13*) at 5 hpi (*n* ≥ 10, one-way ANOVA).

To verify whether Ripk3 plays a role in the infectious inflammatory response, we treated zebrafish larvae with LPS, a gram-negative bacterial component. WT larvae were first injected with LPS or PBS (as control) at 3 dpf in the yolk and collected at different time points to optimize the most robust infectious inflammation response (Fig. 5A). We found that the LPS-injected larvae showed apparent local neutrophil accumulation at 2 hpi compared with the control group (Fig. S2A, B). The accumulation of neutrophils peaked at 5 hpi and started to decrease afterward (Fig. S2A, B). We thus detected the expression of pro-inflammatory cytokines in LPS- and PBS-injected groups at 5 hpi. The pro-inflammatory factors in WT zebrafish were generally upregulated after the LPS challenge (Fig. S2C). The above data demonstrated that the microinjection of LPS into 3 dpf zebrafish larvae could successfully induce a strong infectious inflammatory response.

Next, we treated the *ripk3*-deficient and WT larvae with LPS and collected them at 2, 5, and 8 hpi to detect their neutrophil enrichment status (Fig. 5A). Interestingly, although WT larvae exhibited a robust accumulation of neutrophils, the neutrophils of *ripk3*-deficient larvae remained unchanged in the yolk region (Fig. 5B–D). Thus, this phenomenon demonstrated that Ripk3 is essential for the LPS-induced accumulation of neutrophils in inflammatory responses.

We also detected the expression levels of cytokines at 5 hpi. Upon LPS treatment, the expression of pro-inflammatory cytokines *il1b* and *il6* were markedly upregulated in both WT and *ripk3*-deficient larvae, although the upregulated level in *ripk3*-deficient larvae was not as high as in WT (Fig. 5E), suggesting that the *ripk3* deficiency might weaken the pro-inflammatory process. However, LPS induced only slight changes of *cxcl8a* expression in WT and *ripk3*-deficient larvae (Fig. 5E), suggesting that *cxcl8a* might not be critical for LPS induced inflammation responses. For the anti-inflammatory cytokines, *il4* and *il13* were significantly downregulated in WT larvae upon LPS treatment, whereas the LPS induced downregulation of the two cytokines was blocked in the challenged *ripk3*-deficient larvae (Fig. 5F). However, the expression of *il10* remained unchanged in WT larvae but upregulated robustly in *ripk3*-deficient larvae after LPS induction (Fig. 5F). Thus, during the LPS challenge process, *ripk3*-deficient larvae showed the attenuation of pro-inflammatory cytokine expression and the maintenance of relatively high anti-inflammatory cytokine levels.

Taken together, we demonstrated that in infection-induced inflammation, the *ripk3* deficiency could block neutrophil accumulation, prevent pro-inflammatory cytokine over-production, and maintain relatively high anti-inflammatory cytokine levels, suggesting that *ripk3* perturbation might diminish inflammation in infectious disorders.

### Targeting Ripk3 could inhibit LPS-induced inflammation

To evaluate whether Ripk3 could serve as a candidate target for treating inflammatory responses, we applied Ripk3 inhibitors, namely GSK872, GSK840, GSK843, and RIPK3-IN-1, to WT larvae and injected them with LPS. The concentrations of Ripk3 inhibitors were tested with 2 dpf WT larvae, in which the teratogenic concentrations were identified (Table 1). The final treatment concentrations were selected as around half the teratogenic concentrations (Table 1).

**Fig. 6:**
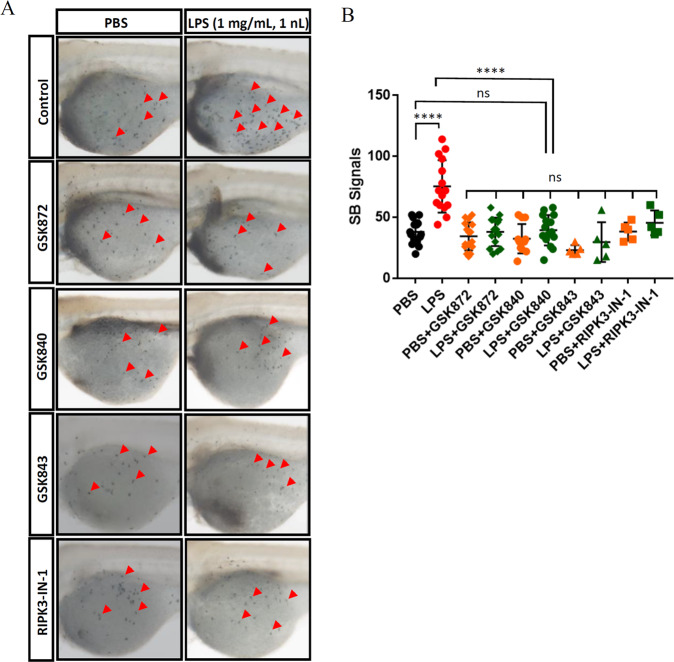
Targeting Ripk3 could inhibit LPS-induced inflammation. **A** Attenuated LPS-induced neutrophil accumulation upon Ripk3 inhibition. The WT larvae were pre-treated with Ripk3 inhibitors and then injected with LPS at 3 dpf. The neutrophil accumulation (red arrows pointed) was significantly inhibited by Ripk3 inhibitors at 5 hpi. **B** Quantification of (**A**) (*n* ≥ 5, one-way ANOVA).

We next investigated whether the LPS-induced SB+ neutrophil accumulation could be blocked by RIPK3 inhibitors. When treated with these inhibitors, the PBS-injected groups showed no significant difference compared with the non-treated control group, indicating that these inhibitors would not affect the basal development of neutrophils (Fig. 6A, B). When LPS injection was performed along with the treatment of RIPK3 inhibitors, no significant difference could be found between the LPS- and PBS-injected larvae in each group (Fig. 6A, B), indicating that the LPS-induced neutrophil enrichment was abolished by inhibiting RIPK3. Therefore, the results validated the essential role of Ripk3 in neutrophil-related inflammatory responses and also implied that targeting RIPK3 to diminish inflammation could have potential application in clinical treatments.

## Discussion

In the present research, we generated a *ripk3*-deficient zebrafish line and investigated the role of Ripk3 in inflammatory responses. The results showed that *ripk3*-deficient zebrafish could serve as an animal model to further monitor inflammatory-related diseases with impairment of neutrophil responses. As we demonstrated that targeting *ripk3* could block neutrophil accumulation in the model, we believe that Ripk3 could be a potential therapeutic target to treat neutrophil-associated inflammatory diseases.

Since leukocytes are the main effector cells involved in inflammation, we first sought to detect whether the deficiency of *ripk3* affects hematopoiesis in homeostatic status, especially the development of leukocytes. It was found that myeloid, lymphoid, and erythroid hematopoiesis were all unaltered in the *ripk3*-deficient larvae. In mice, RIPK1, another member of the RIPK family, was found to regulate embryonic hematopoiesis [27]. When the RIPK1 suppressors, LUBAC and Caspase-8, are absent, RIPK3 could prevent RIPK1 from inducing lethal hematopoietic defects [27]. Therefore, only the triple mutations of *Ripk3*, *Caspase-8*, and *Hoil-1* (LUBAC component) would trigger severe RIPK1-dependent hematopoietic defects [27], while a single mutation of the *Ripk3* gene was not sufficient to affect hematopoiesis. In another study, bone-marrow-specific knockout of the *Ripk3* gene induced higher cell death rates of the bone-marrow nucleated cells [28]. However, the overall hematopoietic lineages exhibited no defect, probably due to the stronger expansion activities of hematopoietic stem/progenitor cells [28]. Combined with our research in zebrafish, it could be concluded that the deficiency of Ripk3 is not sufficient to affect homeostatic hematopoiesis.

Subsequently, to characterize the roles of Ripk3 in non-infectious inflammatory responses, we induced caudal fin fold injury in the *ripk3*-deficient larvae. In the caudal fin fold transected larvae, both *ripk3*-deficient and WT individuals exhibited similar neutrophil recruitment and cytokine expression. It was thus suggested that the physical, non-infectious inflammatory responses were likely to be independent of Ripk3. During tissue injuries, damage-associated molecular pattern proteins (DAMPs) were released [29]. DAMPs can activate classical pattern recognition receptors (PRRs) [30]. Besides, DAMPs can also be sensed by various non-PRR receptors, including the receptor for advanced glycation end products (RAGE) [31], triggering receptors expressed on myeloid cells (TREMs) [32] and N-formyl peptide receptors (FPRs) [33]. Noteworthily, RAGE, TREMs, and FPRs are all expressed on neutrophils [29]. Therefore, the highly complicated DAMP-related inflammatory pathways, triggering neutrophil recruitment to the injured caudal fin fold, are not likely to depend on Ripk3 function in zebrafish.

To characterize the roles of Ripk3 in infectious inflammatory responses, we microinjected the *ripk3*-deficient larvae with LPS. Interestingly, it was found that the deficiency of *ripk3* abolished the enrichment of neutrophils in the injection site. To decipher the underlying mechanisms of this phenomenon, the expression of pro-inflammatory cytokines was detected, and *il1b* was found to be expressed significantly higher in LPS-injected WT larvae. The well-known pro-inflammatory cytokine IL-1β is mainly released by macrophages [24] and neutrophils are recruited by IL-1β during inflammation [34]. In mammals, it has been reported that RIPK3 plays indispensable roles in activating the NLRP3 inflammasome, which mediates the maturation and secretion of IL-1β [15]. Therefore, RIPK3 might play conserved roles in inducing the production of IL-1β in both zebrafish and mammals. The insufficiency of IL-1β production, probably in macrophages, might be responsible for the impairment of neutrophil enrichment.

The expression levels of anti-inflammatory cytokines (*il4*, *il10*, and *il13*) were also highly expressed in *ripk3*-deficient larvae during LPS-induced inflammation. IL-4 could dramatically inhibit the secretion of IL-1β, IL-6, and TNF-α by monocytes [35]. IL-13 could also suppress the production of cytokines such as IL-1β, IL-6, and IL-8 by LPS-treated monocytes [36]. IL-10 is the most important anti-inflammatory cytokine that could inhibit the pro-inflammatory cytokines derived by monocytes, neutrophils, and natural killer cells [25]. Therefore, the upregulation of these anti-inflammatory cytokines might exert their functions by suppressing the release of pro-inflammatory cytokines, thus inhibiting the recruitment of neutrophils. The relationship between Ripk3 and these anti-inflammatory cytokines has barely been reported, it is worth further investigation and might reveal novel regulation networks in inflammatory responses.

Since the *ripk3*-deficient larvae exhibited attenuated neutrophil accumulation upon LPS infections, this zebrafish line could serve as a disease model to simulate diseases in which neutrophil enrichment was dysfunctional. For example, sepsis is a syndrome secondary to infections in which the neutrophil migration toward infection sites was severely impaired [5]. Therefore, when a bacterial or fungal infection was induced in *ripk3*-deficient zebrafish, the defected neutrophil responses would probably trigger sepsis-like disorders. It is worth developing more disease models based on the *ripk3*-deficient zebrafish, as the zebrafish model is quite suitable for drug evaluation and screening.

On the other hand, since neutrophil roles in different diseases vary largely, we also sought the possible applications of targeting Ripk3 in treating neutrophilia-related diseases. When we applied Ripk3 inhibitors to LPS-treated larvae, the enrichment of neutrophils in the injection site was strongly inhibited by these inhibitors. Therefore, targeting Ripk3 could also serve as a possible clinical treatment to contain diseases with neutrophilic inflammation. Diseases such as Alzheimer’s disease, epilepsy, type II diabetes, systemic lupus erythematosus, and atherosclerosis are all associated with local accumulation of pro-inflammatory neutrophils [8], and thus might be potential applications for Ripk3-targeted treatments.

In summary, our study revealed an indispensable role of Ripk3 in LPS-induced inflammatory responses, accumulating neutrophils in the inflammation site. The *ripk3*-deficient zebrafish line could serve as a disease model of impaired neutrophil enrichment in infectious inflammation responses. In addition, targeting Ripk3 could attenuate the accumulation of neutrophils to the inflammation sites, providing valuable insights into developing relevant therapeutic strategies for diminishing inflammation in clinical applications.

## Materials and methods

### Zebrafish maintenance

AB strain WT zebrafish were maintained according to standard protocols [37]. The adult zebrafish were maintained in a recirculating system at 28 °C under a 14/10 h light/dark cycle. Before the experiment, zebrafish were intercrossed in a male-to-female ratio of 1:2, and their embryos were incubated in egg water containing 0.003% 1-phenyl-2-thiourea (PTU).

### Generation of the *ripk3*-deficient line

Mutagenesis was performed with CRISPR/Cas9 technology as described [38, 39]. The *ripk3* gRNA (5′-CCGTGTGCTCTCGCCCTCC-3′) was synthesized with T7 RNA polymerase (Thermo Fisher, USA), mixed with Cas9 protein (New England Biolabs, USA), and injected into 1-cell stage WT zebrafish embryos. The embryos were raised to adulthood as F0 generation and crossed with WT zebrafish to obtain F1 generation embryos with *ripk3* deficiency. Finally, an F1 founder line with 17 bp deletion of the *ripk3* gene was obtained and applied in further experiments.

### WISH

The *mfap4*, *rag1*, and *βe1* probes labeled with Digoxigenin (Roche, Switzerland) were transcribed in vitro with T7 RNA polymerase (Thermo Fisher, USA). The 4% paraformaldehyde (PFA, Sigma-Aldrich, USA) fixed zebrafish larvae were hybridized with either a *rag1* or *βe1* probes according to the standard protocol [40]. For the WISH of *mfap4* probe, we used maleic acid buffer in antibody blocking and washing, for a better signal-to-noise ratio [41].

### RNA extraction and Q-PCR

For each Q-PCR sample, at least 10 larvae were collected as a pool. The total RNA of each sample was isolated using TRIzol reagent (Thermo Fisher, USA), and then the cDNA was generated using a HiScript II Q Select RT SuperMix kit (Vazyme, China) according to the manufacturer’s instructions. Three samples were applied in each Q-PCR group. Q-PCR was performed using a SYBR Green PCR Core Reagent kit on a LightCycler 96 system (Roche, Switzerland). Fold changes were determined by the ΔΔ comparative threshold method. Primers are detailed in Table S1.

In addition, for WT and *ripk3*-deficient larvae, a common Q-PCR forward primer (co_FP) and a reverse primer (RP) were designed to detect the expression of *ripk3*. On the other hand, a WT-specific forward primer (wt_FP) and a mutant-specific forward primer (mut_FP) were respectively designed to detect the expression of WT or mutant form of *ripk3* mRNA.

### LPS microinjection and RIPK3 inhibitor treatment

To establish the inflammatory model, 3 dpf zebrafish larvae were anesthetized with 0.02% tricaine and yolk-microinjected with 1 nL LPS (1 mg/mL) per larva on an agarose plate as reported previously [42]. PBS-injected larvae were used as the control group.

Four different Ripk3 inhibitors (GSK872, GSK840, GSK843, and RIPK3-IN-1) [43, 44] were purchased from MedChemExpress, USA. The concentrations of these inhibitors used were listed in Table 1. When treated with Ripk3 inhibitors, WT larvae were soaked in egg water with inhibitors from 2 dpf. The larvae were injected with PBS or LPS at 3 dpf and further soaked in egg water with inhibitors until collected.

**Table 1:** The concentrations of RIPK3 inhibitors used.

RIPK3 inhibitor	Teratogenic concentration	Experimental concentration
GSK872	100 μM	30 μM
GSK840	20 μM	10 μM
GSK843	5 μM	2.5 μM
RIPK3-IN-1	30 μM	20 μM

The used concentrations of RIPK3 inhibitors were listed. The teratogenic concentrations represent the minimal concentrations that would induce larval deformity, while the experimental concentrations represent the final applied concentrations in this study.

### Caudal fin fold injury

The 3 dpf larvae were first anesthetized with 0.02% tricaine. Then, their caudal fin folds were transected with a disposable syringe needle on a sterile plastic Petri dish to induce the injury [45].

### SB staining

SB is a lipid stain that strongly favors the staining of the granules of granulocytes [46], which are all neutrophils in 3 dpf zebrafish larvae. Therefore, using SB staining, we could specifically mark the neutrophils and study their behaviours. After LPS microinjection or caudal fin fold injury, 3 dpf larvae were fixed with 4% PFA for 2 h at room temperature, washed twice with PBST, and then stained with SB solution for 30 min as described [46]. After that, the larvae were washed extensively in 70% ethanol, and their SB positive signals were observed by a Zeiss stereomicroscope. The SB signals (black coloured) were counted manually, with the *Z*-plane of every signal carefully examined to avoid miss counting.

### Statistical analysis

The differences between categorical variables were analyzed by Fisher’s exact tests. The continuous variables were analyzed using Student’s t-test or one-way analysis of variance (ANOVA), depending on whether the comparisons were between two groups or among multiple groups, respectively. The figures were expressed as mean ± SEM from at least three independent experiments with duplicate samples and analyzed with the statistical software GraphPad Prism 7.0. The values were considered statistically significant when *P* < 0.05 (ns indicates not significant; * indicates *p* < 0.05; ** indicates *p* < 0.01; *** indicates *p* < 0.001, and **** indicates *p* < 0.0001) and shown as means ± SEM.

## Supplementary information

Supplementary figures and table

## Data Availability

Data sharing is not applicable as no dataset was generated.
