# Supplementary material for: Loss of Ripk3 attenuated neutrophil accumulation in a lipopolysaccharide-induced zebrafish inflammatory model
Source: Cell Death Discov. 2022 Feb 26;8:88. doi: 10.1038/s41420-022-00891-z (PMC8882176; doi:10.1038/s41420-022-00891-z)
Supplement: Supplementary file 1 — Supplementary figures and table [file 41420_2022_891_MOESM1_ESM.pdf]

Figure. S1

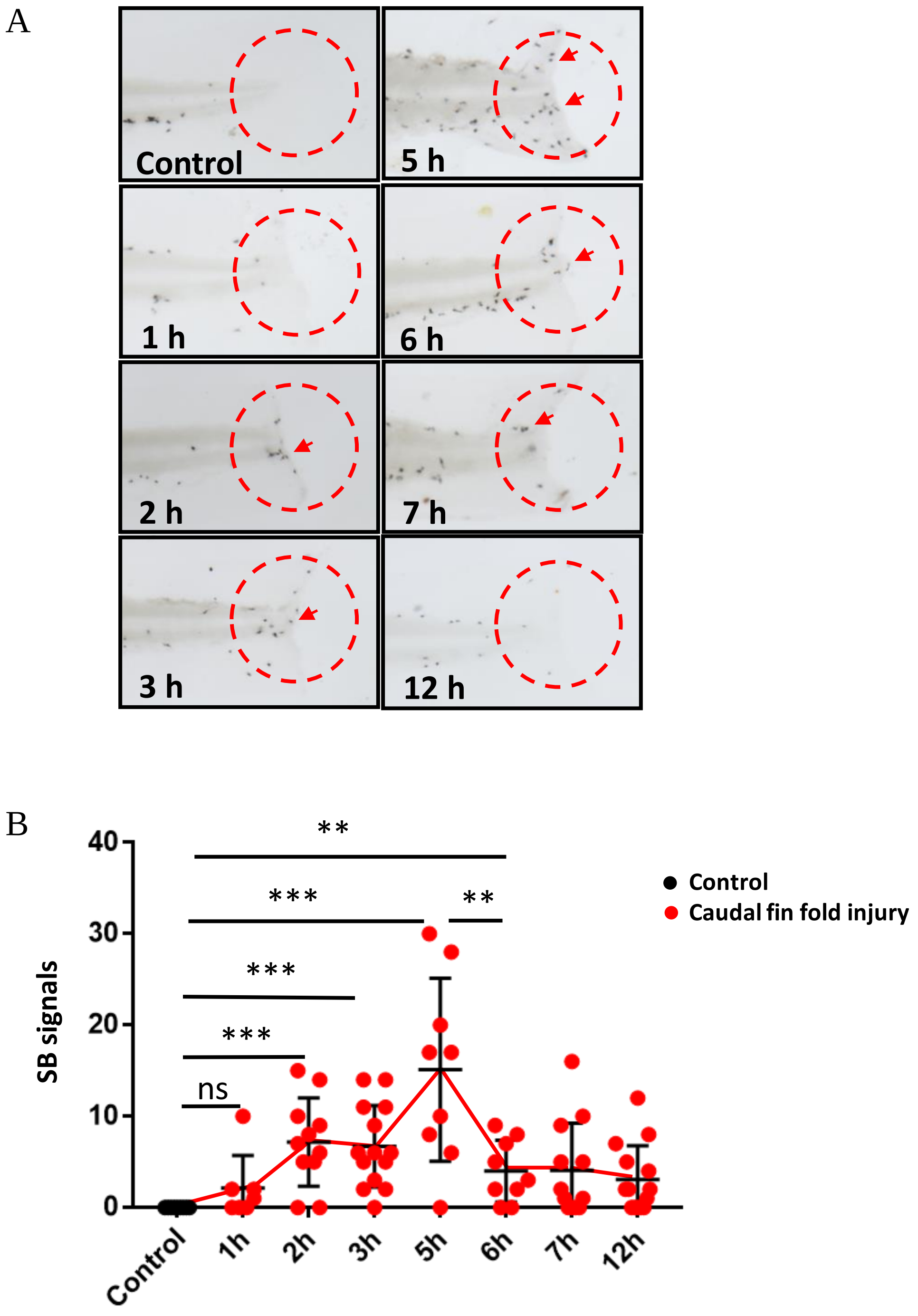

Figure S1. Caudal fin fold injury induced strongest inflammation at 5 hpi. (A) Optimizing the strongest non-infectious inflammation. SB staining showed the neutrophil accumulation status at various time points post injury. (B) Quantification of (A) (means±SEM, n≥10. One-way ANOVA: ns indicates not significant; ** indicates $p<0.01$; *** indicates $p<0.001$).

Figure. S2

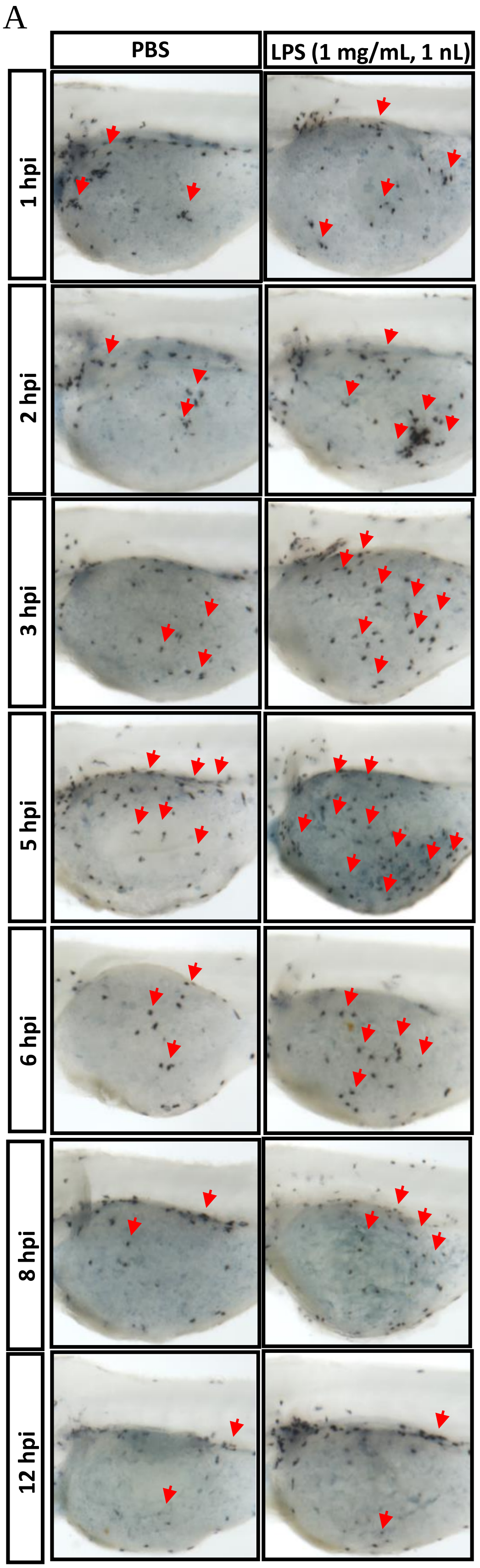

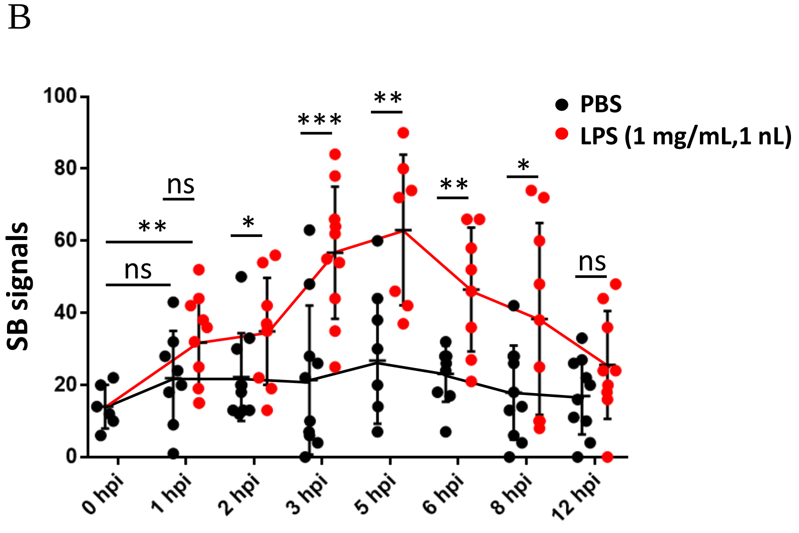

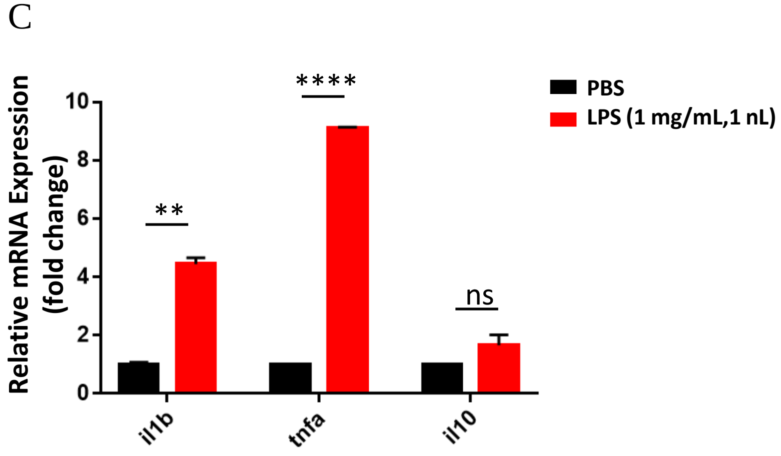

Figure S2. LPS induced strongest inflammation at 5 hpi. (A) Optimizing the strongest LPS-induced inflammation. SB staining showed the neutrophil accumulation status at various time points post LPS injection. (B) Quantification of (A) (means±SEM, n≥10. Student’s t-test: ns indicates not significant; * indicates p<0.05; ** indicates p<0.01; *** indicates p<0.001). (C) LPS-induced cytokines expression. Q-PCR revealed the expression levels of *il1b*, *tnfa*, and *il10* in PBS- and LPS-injected WT larvae at 5 hpi (means±SEM, n≥10. Student’s t-test: ns indicates not significant; ** indicates p<0.01; **** indicates p<0.0001).

Table. S1 The list of qRT-PCR primers

gene	Primer sequences (5′-3′)
wt_FP	TGGCTGCTCCTCCGCCG
mut_FP	GCTGCAGAGAT TGTGCTCTCG
co_FP	TTTGTCCCGAGTGGCTGAAA
*ripk3*_qRP	GACTGGACTGAACACCGCT
*Tnfα*_FP	GCGCTTTTCTGAATCCTACG
*Tnfα*_RP	TGCCCAGTCTGTCTCCTTCT
*il1b*_FP	CTGGAGATGTGGACTTCGCA
*il1b*_RP	TCACGCTCTTGGATGACGTT
*il6*_FP	GGCATTTGAAGGGGTCAGGA
*il6*_RP	GCGTTAGACATCTTTCCGTGC
*cxcl8a*_FP	AACAGAAAGCCGACGCATTG
*cxcl8a*_RP	TGTCATCAAGGTGGCAATGAT
*il4*_FP	CAGCATATACCGGGACTGGAA
*il4*_RP	GATAATGGCAGCATGCTTTGGT
*il10*_FP	CGGGATATGGTGAAATGCAAGA
*il10*_RP	AGAGCAAATCAAGCTCCCCC
*il13*_FP	CCCCAAAAGAGACAAAGGCAA
*il13*_RP	TCACACTTCAGGCCACTTCC
